# 5-Fluoro-6′*H*,7′*H*,8′*H*-spiro­[indoline-3,7′-pyrano[3,2-*c*:5,6-*c*′]di-1-benzopyran]-2,6′,8′-trione

**DOI:** 10.1107/S1600536812006332

**Published:** 2012-02-17

**Authors:** Abdulrahman I. Almansour, Raju Suresh Kumar, Natarajan Arumugam, P. Devi Shree, J. Suresh

**Affiliations:** a Department of Chemistry, College of Sciences, King Saud University, PO Box 2455, Riyadh 11451, Saudi Arabia; b Department of Physics, Madura College, Madurai 625 011, India

## Abstract

In the title compound, C_26_H_12_FNO_6_, the central pyran ring and both benzopyran systems are nonplanar, having total puckering amplitudes of 0.139 (2), 0.050 (1) and 0.112 (2) Å, respectively. The central pyran ring adopts a boat conformation. The crystal structure is stabilized by C—H⋯O, N—H⋯O, N—H⋯F and C—H⋯π inter­actions.

## Related literature

For the background to benzopyran derivatives, see: Martin & Critchlow (1999 ▶); Teague & Davis (1999 ▶); Joshi & Jain (1985 ▶); Ninamiya (1980 ▶); Kobayashi & Matsuda (1970 ▶). For hydrogen-bonding motifs, see: Bernstein *et al.* (1995 ▶).
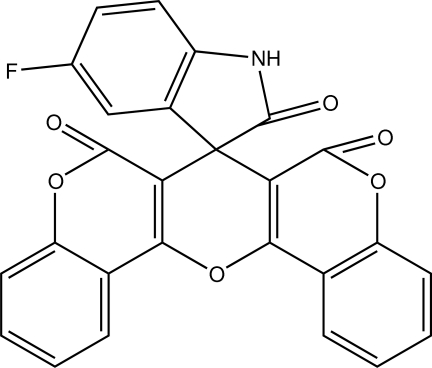


## Experimental

### 

#### Crystal data

C_26_H_12_FNO_6_
*M* *_r_* = 453.37
Triclinic, 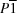

*a* = 7.8262 (1) Å
*b* = 10.9278 (1) Å
*c* = 12.4067 (2) Å
α = 113.374 (1)°
β = 94.922 (1)°
γ = 100.295 (1)°
*V* = 943.77 (2) Å^3^
*Z* = 2
Mo *K*α radiation
μ = 0.12 mm^−1^
*T* = 293 K
0.23 × 0.21 × 0.18 mm

#### Data collection

Bruker Kappa APEXII diffractometer
Absorption correction: multi-scan (*SADABS*; Sheldrick, 1996 ▶) *T*_min_ = 0.973, *T*_max_ = 0.978
15720 measured reflections
5504 independent reflections
4486 reflections with *I* > 2σ(*I*)
*R*_int_ = 0.023

#### Refinement

*R*[*F* ^2^ > 2σ(*F* ^2^)] = 0.045
*wR*(*F* ^2^) = 0.124
*S* = 1.04
5504 reflections
311 parameters
1 restraint
H atoms treated by a mixture of independent and constrained refinement
Δρ_max_ = 0.46 e Å^−3^
Δρ_min_ = −0.29 e Å^−3^

### 

Data collection: *APEX2* (Bruker, 2004 ▶); cell refinement: *SAINT* (Bruker, 2004 ▶); data reduction: *SAINT*; program(s) used to solve structure: *SHELXS97* (Sheldrick, 2008 ▶); program(s) used to refine structure: *SHELXL97* (Sheldrick, 2008 ▶); molecular graphics: *PLATON* (Spek, 2009 ▶); software used to prepare material for publication: *SHELXL97*.

## Supplementary Material

Crystal structure: contains datablock(s) global, I. DOI: 10.1107/S1600536812006332/tk5055sup1.cif

Structure factors: contains datablock(s) I. DOI: 10.1107/S1600536812006332/tk5055Isup2.hkl

Supplementary material file. DOI: 10.1107/S1600536812006332/tk5055Isup3.cml

Additional supplementary materials:  crystallographic information; 3D view; checkCIF report

## Figures and Tables

**Table 1:** Hydrogen-bond geometry (Å, °) *Cg*1 is the centroid of the C41–C46 ring.

*D*—H⋯*A*	*D*—H	H⋯*A*	*D*⋯*A*	*D*—H⋯*A*
C45—H45⋯O5^i^	0.93	2.50	3.2564 (18)	139
C22—H22⋯O6^ii^	0.93	2.46	3.2205 (17)	139
C66—H66⋯O6^ii^	0.93	2.50	3.2921 (17)	144
C64—H64⋯O3^iii^	0.93	2.49	3.2641 (18)	141
C25—H25⋯*Cg*1^iv^	0.93	2.65	3.5482 (17)	163
N1—H1⋯F1^v^	0.86 (2)	2.15 (2)	2.810 (2)	134 (2)
N1—H1⋯O5^i^	0.86 (2)	2.51 (2)	3.208 (2)	140 (2)

Symmetry codes: (i) 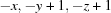; (ii) 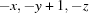; (iii) 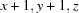; (iv) 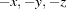; (v) 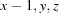.

**Fig. 1.**
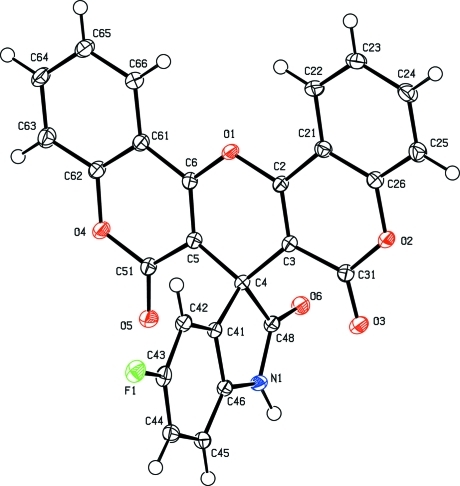
The molecular structure of (I), showing 50% probability displacement ellipsoids and the atom-numbering scheme.

**Fig. 2.**
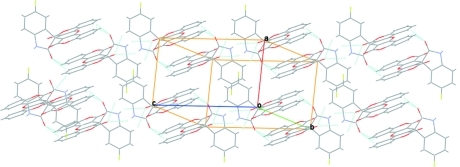
A packing diagram for (I).

## supplementary crystallographic information

### Comment

Benzopyran is a structural motif observed in many biologically active natural products and it plays an important role in binding to various biopolymers (Martin & Critchlow, 1999; Teague & Davis, 1999). Spiro indoles are also known for their broad spectrum of biological activities (Joshi & Jain, 1985). Of the various spiro indoles, the spiro[indole-pyran] system has attracted attention owing to its interesting pharmacological properties (Ninamiya, 1980; Kobayashi & Matsuda, 1970). The biological importance of these heterocycles in conjunction with our research interests, prompted us to synthesize and report the X-ray structure of the title compound, (I).

In (I), Fig. 1, the central pyrano ring A (O1–C6) and both the benzopyran rings B (C5/C6/C61–C66/O4/C51), C (C3/C3/C21–C26/O2/C31) are non-planar, having total puckering amplitudes, Q_T_, of 0.139 (2), 0.050 (1) and 0.112 (2) Å, respectively. The central pyrano ring adopts a boat conformation [Φ = 357.6 (6)° and θ = 109.0 (6)°]. In the indolin-2-one system, the benzene and pyrrole rings are individually planar and make a dihedral angle of 2.20 (1)°. The indoline-2-one system is in a perpendicular configuration with respect to the pyrano ring, as can be seen from the dihedral angle [89.83 (2)°]. The sum of the angles at atom N1 of the indolin-2-one moiety is in accordance with *sp*^2^-hybridization [359.41 (2)°].

The N1—H1···O5 hydrogen bonds connect two centrosymmetrically related molecules and generate the graph set motif *R*_2_^2^(14) (Bernstein *et al.*, 1995). The centrosymmetric dimers are interconnected into zig-zag linear chain of C—H···O hydrogen bonds and the molecules form a layered structure (Fig. 2). In addition, there is a weak C—H···π interaction, *viz.* C25—H25···*Cg*1 (*Cg*1 is the centroid of the ring C41–C46; symmetry codes are given in Table 1).

### Experimental

A mixture of 5-fluoroindoline-2,3-dione (0.100 g, 0.60 mmol), 4-hydroxy-2*H*-chromen-2-one (0.194 g, 1.20 mmol), and paratoluene sulfonic acid (0.114 g, 0.60 mmol) were dissolved in 5 ml of ethanol:water (1:1 *v*/*v*) and refluxed for 2 h. After completion of the reaction as evident from TLC, the precipitated solid was filtered and washed with water to afford the product which was recrystallized from ethanol to provide colourless crystals. Yield 72%, m.p. 541–543 K.

### Refinement

The N1—H atom was located in a difference map and refined with an N—H distance restraint of 0.86±0.01 Å. The C-bound H atoms were placed at calculated positions and allowed to ride on their carrier atoms with C—H = 0.93 Å,and with *U*_iso_ = 1.2*U*_eq_(C).

### Crystal data

**Table:**

C_26_H_12_FNO_6_	*Z* = 2
*M**_r_* = 453.37	*F*(000) = 464
Triclinic, *P*1	*D*_x_ = 1.595 Mg m^−^^3^
Hall symbol: -P 1	Mo *K*α radiation, λ = 0.71073 Å
*a* = 7.8262 (1) Å	Cell parameters from 2000 reflections
*b* = 10.9278 (1) Å	θ = 2–31°
*c* = 12.4067 (2) Å	µ = 0.12 mm^−^^1^
α = 113.374 (1)°	*T* = 293 K
β = 94.922 (1)°	Block, colourless
γ = 100.295 (1)°	0.23 × 0.21 × 0.18 mm
*V* = 943.77 (2) Å^3^	

### Data collection

**Table:**

Bruker Kappa APEXII diffractometer	5504 independent reflections
Radiation source: fine-focus sealed tube	4486 reflections with *I* > 2σ(*I*)
Graphite monochromator	*R*_int_ = 0.023
Detector resolution: 0 pixels mm^-1^	θ_max_ = 30.1°, θ_min_ = 1.8°
ω and φ scans	*h* = −11→11
Absorption correction: multi-scan (*SADABS*; Sheldrick, 1996)	*k* = −15→15
*T*_min_ = 0.973, *T*_max_ = 0.978	*l* = −17→12
15720 measured reflections	

### Refinement

**Table:**

Refinement on *F*^2^	Primary atom site location: structure-invariant direct methods
Least-squares matrix: full	Secondary atom site location: difference Fourier map
*R*[*F*^2^ > 2σ(*F*^2^)] = 0.045	Hydrogen site location: inferred from neighbouring sites
*wR*(*F*^2^) = 0.124	H atoms treated by a mixture of independent and constrained refinement
*S* = 1.04	*w* = 1/[σ^2^(*F*_o_^2^) + (0.0602*P*)^2^ + 0.4439*P*] where *P* = (*F*_o_^2^ + 2*F*_c_^2^)/3
5504 reflections	(Δ/σ)_max_ < 0.001
311 parameters	Δρ_max_ = 0.46 e Å^−^^3^
1 restraint	Δρ_min_ = −0.29 e Å^−^^3^

### Special details

**Table:**

Geometry. All e.s.d.'s (except the e.s.d. in the dihedral angle between two l.s. planes) are estimated using the full covariance matrix. The cell e.s.d.'s are taken into account individually in the estimation of e.s.d.'s in distances, angles and torsion angles; correlations between e.s.d.'s in cell parameters are only used when they are defined by crystal symmetry. An approximate (isotropic) treatment of cell e.s.d.'s is used for estimating e.s.d.'s involving l.s. planes.
Refinement. Refinement of *F*^2^ against ALL reflections. The weighted *R*-factor *wR* and goodness of fit *S* are based on *F*^2^, conventional *R*-factors *R* are based on *F*, with *F* set to zero for negative *F*^2^. The threshold expression of *F*^2^ > σ(*F*^2^) is used only for calculating *R*-factors(gt) *etc*. and is not relevant to the choice of reflections for refinement. *R*-factors based on *F*^2^ are statistically about twice as large as those based on *F*, and *R*-factors based on ALL data will be even larger.

### Fractional atomic coordinates and isotropic or equivalent isotropic displacement parameters (Å^2^)

**Table:**

	*x*	*y*	*z*	*U*_iso_*/*U*_eq_	
H1	−0.122 (2)	0.3850 (19)	0.4085 (15)	0.025 (5)*	
C2	0.12458 (17)	0.34311 (13)	−0.00317 (11)	0.0140 (2)	
C3	0.05623 (17)	0.31770 (13)	0.08465 (11)	0.0135 (2)	
C4	0.09926 (16)	0.42071 (13)	0.21373 (11)	0.0128 (2)	
C5	0.18729 (17)	0.55860 (13)	0.21847 (11)	0.0139 (2)	
C6	0.24834 (17)	0.57319 (13)	0.12440 (11)	0.0139 (2)	
C21	0.09810 (17)	0.23914 (13)	−0.12381 (11)	0.0148 (2)	
C22	0.17434 (19)	0.25796 (14)	−0.21611 (12)	0.0172 (3)	
H22	0.2407	0.3436	−0.2034	0.021*	
C23	0.15001 (19)	0.14852 (15)	−0.32576 (12)	0.0191 (3)	
H23	0.2016	0.1604	−0.3868	0.023*	
C24	0.0486 (2)	0.01981 (15)	−0.34611 (12)	0.0199 (3)	
H24	0.0344	−0.0533	−0.4203	0.024*	
C25	−0.03060 (19)	0.00031 (14)	−0.25711 (12)	0.0194 (3)	
H25	−0.1002	−0.0847	−0.2711	0.023*	
C26	−0.00412 (17)	0.11020 (14)	−0.14632 (12)	0.0156 (3)	
C31	−0.05619 (17)	0.18354 (14)	0.05589 (12)	0.0152 (2)	
C41	0.21280 (17)	0.37727 (13)	0.29163 (11)	0.0130 (2)	
C42	0.37794 (17)	0.34883 (13)	0.28347 (12)	0.0155 (3)	
H42	0.4387	0.3545	0.2238	0.019*	
C43	0.44766 (17)	0.31133 (14)	0.36932 (12)	0.0164 (3)	
C44	0.36544 (18)	0.30651 (14)	0.46197 (12)	0.0168 (3)	
H44	0.4203	0.2841	0.5188	0.020*	
C45	0.19881 (18)	0.33555 (13)	0.46972 (12)	0.0156 (3)	
H45	0.1405	0.3334	0.5314	0.019*	
C46	0.12383 (17)	0.36764 (13)	0.38189 (11)	0.0134 (2)	
C48	−0.07059 (17)	0.42709 (13)	0.27287 (11)	0.0140 (2)	
C51	0.21637 (17)	0.67890 (13)	0.33197 (12)	0.0157 (3)	
C61	0.34501 (17)	0.70292 (14)	0.13272 (12)	0.0151 (2)	
C62	0.37581 (17)	0.81431 (14)	0.24372 (12)	0.0162 (3)	
C63	0.47226 (19)	0.94335 (14)	0.26308 (13)	0.0196 (3)	
H63	0.4916	1.0166	0.3377	0.024*	
C64	0.53871 (19)	0.96018 (15)	0.16875 (13)	0.0198 (3)	
H64	0.6045	1.0455	0.1803	0.024*	
C65	0.50796 (19)	0.85014 (15)	0.05598 (13)	0.0195 (3)	
H65	0.5525	0.8632	−0.0068	0.023*	
C66	0.41184 (18)	0.72236 (14)	0.03748 (12)	0.0173 (3)	
H66	0.3914	0.6496	−0.0375	0.021*	
N1	−0.04170 (15)	0.39656 (12)	0.36839 (10)	0.0140 (2)	
O1	0.22685 (13)	0.46733 (10)	0.01416 (8)	0.0159 (2)	
O2	−0.08257 (13)	0.08560 (10)	−0.05945 (8)	0.0167 (2)	
O3	−0.12795 (14)	0.15262 (10)	0.12640 (9)	0.0195 (2)	
O4	0.31252 (13)	0.80145 (10)	0.33995 (9)	0.0180 (2)	
O5	0.16406 (14)	0.67882 (10)	0.42086 (9)	0.0194 (2)	
O6	−0.20150 (13)	0.45589 (10)	0.23875 (9)	0.0168 (2)	
F1	0.60614 (11)	0.27654 (9)	0.36098 (8)	0.02124 (19)	

### Atomic displacement parameters (Å^2^)

**Table:**

	*U*^11^	*U*^22^	*U*^33^	*U*^12^	*U*^13^	*U*^23^
C2	0.0134 (6)	0.0131 (6)	0.0156 (6)	0.0008 (4)	0.0019 (4)	0.0072 (5)
C3	0.0134 (6)	0.0124 (6)	0.0142 (6)	0.0009 (4)	0.0018 (4)	0.0060 (5)
C4	0.0124 (5)	0.0129 (6)	0.0132 (5)	0.0006 (4)	0.0022 (4)	0.0066 (5)
C5	0.0134 (6)	0.0135 (6)	0.0142 (6)	0.0013 (4)	0.0013 (4)	0.0062 (5)
C6	0.0128 (6)	0.0130 (6)	0.0145 (6)	0.0012 (4)	0.0013 (4)	0.0054 (5)
C21	0.0159 (6)	0.0150 (6)	0.0139 (6)	0.0036 (5)	0.0018 (4)	0.0065 (5)
C22	0.0205 (6)	0.0170 (6)	0.0165 (6)	0.0032 (5)	0.0028 (5)	0.0101 (5)
C23	0.0233 (7)	0.0214 (7)	0.0144 (6)	0.0047 (5)	0.0043 (5)	0.0095 (5)
C24	0.0264 (7)	0.0169 (6)	0.0139 (6)	0.0040 (5)	0.0003 (5)	0.0051 (5)
C25	0.0226 (7)	0.0147 (6)	0.0178 (6)	0.0015 (5)	−0.0009 (5)	0.0059 (5)
C26	0.0146 (6)	0.0167 (6)	0.0157 (6)	0.0021 (5)	0.0014 (5)	0.0080 (5)
C31	0.0144 (6)	0.0151 (6)	0.0157 (6)	0.0023 (5)	0.0017 (4)	0.0067 (5)
C41	0.0142 (6)	0.0113 (5)	0.0122 (5)	0.0000 (4)	0.0009 (4)	0.0050 (4)
C42	0.0146 (6)	0.0147 (6)	0.0164 (6)	0.0010 (5)	0.0029 (5)	0.0066 (5)
C43	0.0117 (6)	0.0148 (6)	0.0209 (6)	0.0014 (5)	0.0009 (5)	0.0068 (5)
C44	0.0172 (6)	0.0157 (6)	0.0173 (6)	0.0017 (5)	−0.0010 (5)	0.0085 (5)
C45	0.0176 (6)	0.0142 (6)	0.0138 (6)	0.0017 (5)	0.0019 (5)	0.0058 (5)
C46	0.0136 (6)	0.0125 (6)	0.0132 (5)	0.0013 (4)	0.0025 (4)	0.0051 (4)
C48	0.0147 (6)	0.0125 (6)	0.0138 (5)	0.0007 (4)	0.0026 (4)	0.0055 (5)
C51	0.0150 (6)	0.0140 (6)	0.0166 (6)	0.0003 (5)	0.0010 (5)	0.0066 (5)
C61	0.0136 (6)	0.0153 (6)	0.0173 (6)	0.0017 (5)	0.0016 (5)	0.0086 (5)
C62	0.0150 (6)	0.0157 (6)	0.0183 (6)	0.0012 (5)	0.0032 (5)	0.0084 (5)
C63	0.0195 (6)	0.0159 (6)	0.0209 (6)	0.0011 (5)	0.0029 (5)	0.0066 (5)
C64	0.0192 (6)	0.0163 (6)	0.0256 (7)	0.0000 (5)	0.0042 (5)	0.0122 (6)
C65	0.0180 (6)	0.0204 (7)	0.0227 (7)	0.0018 (5)	0.0048 (5)	0.0126 (6)
C66	0.0181 (6)	0.0175 (6)	0.0170 (6)	0.0018 (5)	0.0029 (5)	0.0090 (5)
N1	0.0130 (5)	0.0165 (5)	0.0142 (5)	0.0029 (4)	0.0043 (4)	0.0080 (4)
O1	0.0200 (5)	0.0127 (4)	0.0133 (4)	−0.0008 (4)	0.0042 (3)	0.0055 (4)
O2	0.0186 (5)	0.0141 (4)	0.0153 (4)	−0.0010 (4)	0.0031 (4)	0.0062 (4)
O3	0.0216 (5)	0.0177 (5)	0.0188 (5)	−0.0001 (4)	0.0049 (4)	0.0090 (4)
O4	0.0216 (5)	0.0141 (4)	0.0157 (4)	−0.0015 (4)	0.0047 (4)	0.0055 (4)
O5	0.0223 (5)	0.0189 (5)	0.0149 (4)	0.0007 (4)	0.0045 (4)	0.0063 (4)
O6	0.0150 (4)	0.0181 (5)	0.0186 (5)	0.0037 (4)	0.0022 (3)	0.0092 (4)
F1	0.0125 (4)	0.0252 (4)	0.0288 (5)	0.0054 (3)	0.0035 (3)	0.0138 (4)

### Geometric parameters (Å, º)

**Table:**

C2—C3	1.3555 (18)	C41—C46	1.3968 (18)
C2—O1	1.3712 (16)	C42—C43	1.3880 (18)
C2—C21	1.4448 (18)	C42—H42	0.9300
C3—C31	1.4563 (18)	C43—F1	1.3612 (15)
C3—C4	1.5122 (17)	C43—C44	1.379 (2)
C4—C5	1.5167 (18)	C44—C45	1.3985 (19)
C4—C41	1.5219 (17)	C44—H44	0.9300
C4—C48	1.5716 (18)	C45—C46	1.3862 (18)
C5—C6	1.3535 (18)	C45—H45	0.9300
C5—C51	1.4602 (18)	C46—N1	1.3990 (16)
C6—O1	1.3685 (16)	C48—O6	1.2144 (16)
C6—C61	1.4423 (18)	C48—N1	1.3643 (17)
C21—C26	1.3963 (19)	C51—O5	1.2091 (17)
C21—C22	1.4036 (19)	C51—O4	1.3753 (16)
C22—C23	1.3785 (19)	C61—C62	1.3944 (19)
C22—H22	0.9300	C61—C66	1.4071 (19)
C23—C24	1.399 (2)	C62—O4	1.3785 (16)
C23—H23	0.9300	C62—C63	1.3913 (19)
C24—C25	1.381 (2)	C63—C64	1.384 (2)
C24—H24	0.9300	C63—H63	0.9300
C25—C26	1.3881 (19)	C64—C65	1.402 (2)
C25—H25	0.9300	C64—H64	0.9300
C26—O2	1.3767 (16)	C65—C66	1.3817 (19)
C31—O3	1.2025 (17)	C65—H65	0.9300
C31—O2	1.3751 (16)	C66—H66	0.9300
C41—C42	1.3851 (18)	N1—H1	0.855 (9)
			
C3—C2—O1	123.63 (12)	C43—C42—H42	121.9
C3—C2—C21	122.30 (12)	F1—C43—C44	118.16 (12)
O1—C2—C21	114.06 (11)	F1—C43—C42	117.95 (12)
C2—C3—C31	119.28 (12)	C44—C43—C42	123.88 (12)
C2—C3—C4	122.74 (11)	C43—C44—C45	119.46 (12)
C31—C3—C4	117.91 (11)	C43—C44—H44	120.3
C3—C4—C5	108.11 (10)	C45—C44—H44	120.3
C3—C4—C41	112.55 (10)	C46—C45—C44	117.45 (12)
C5—C4—C41	111.75 (10)	C46—C45—H45	121.3
C3—C4—C48	111.18 (10)	C44—C45—H45	121.3
C5—C4—C48	112.06 (10)	C45—C46—C41	122.00 (12)
C41—C4—C48	101.17 (10)	C45—C46—N1	128.16 (12)
C6—C5—C51	119.03 (12)	C41—C46—N1	109.83 (11)
C6—C5—C4	122.84 (12)	O6—C48—N1	127.29 (13)
C51—C5—C4	118.03 (11)	O6—C48—C4	125.09 (12)
C5—C6—O1	123.61 (12)	N1—C48—C4	107.61 (11)
C5—C6—C61	122.40 (12)	O5—C51—O4	117.02 (12)
O1—C6—C61	113.99 (11)	O5—C51—C5	124.82 (12)
C26—C21—C22	118.97 (12)	O4—C51—C5	118.15 (12)
C26—C21—C2	116.49 (12)	C62—C61—C66	118.90 (12)
C22—C21—C2	124.47 (12)	C62—C61—C6	116.85 (12)
C23—C22—C21	119.45 (13)	C66—C61—C6	124.23 (12)
C23—C22—H22	120.3	O4—C62—C63	116.74 (12)
C21—C22—H22	120.3	O4—C62—C61	121.41 (12)
C22—C23—C24	120.67 (13)	C63—C62—C61	121.85 (13)
C22—C23—H23	119.7	C64—C63—C62	118.45 (13)
C24—C23—H23	119.7	C64—C63—H63	120.8
C25—C24—C23	120.62 (13)	C62—C63—H63	120.8
C25—C24—H24	119.7	C63—C64—C65	120.78 (13)
C23—C24—H24	119.7	C63—C64—H64	119.6
C24—C25—C26	118.62 (13)	C65—C64—H64	119.6
C24—C25—H25	120.7	C66—C65—C64	120.39 (13)
C26—C25—H25	120.7	C66—C65—H65	119.8
O2—C26—C25	116.72 (12)	C64—C65—H65	119.8
O2—C26—C21	121.62 (12)	C65—C66—C61	119.62 (13)
C25—C26—C21	121.66 (13)	C65—C66—H66	120.2
O3—C31—O2	117.67 (12)	C61—C66—H66	120.2
O3—C31—C3	124.30 (12)	C48—N1—C46	112.25 (11)
O2—C31—C3	118.03 (12)	C48—N1—H1	123.1 (13)
C42—C41—C46	120.82 (12)	C46—N1—H1	124.1 (13)
C42—C41—C4	130.10 (12)	C6—O1—C2	117.29 (10)
C46—C41—C4	109.08 (11)	C31—O2—C26	122.11 (11)
C41—C42—C43	116.28 (12)	C51—O4—C62	122.08 (11)
C41—C42—H42	121.9		
			
O1—C2—C3—C31	177.76 (11)	C42—C43—C44—C45	2.5 (2)
C21—C2—C3—C31	−3.55 (19)	C43—C44—C45—C46	0.25 (19)
O1—C2—C3—C4	−5.5 (2)	C44—C45—C46—C41	−2.90 (19)
C21—C2—C3—C4	173.14 (11)	C44—C45—C46—N1	178.11 (12)
C2—C3—C4—C5	13.71 (17)	C42—C41—C46—C45	2.91 (19)
C31—C3—C4—C5	−169.55 (11)	C4—C41—C46—C45	−177.21 (12)
C2—C3—C4—C41	−110.20 (14)	C42—C41—C46—N1	−177.93 (11)
C31—C3—C4—C41	66.54 (15)	C4—C41—C46—N1	1.94 (14)
C2—C3—C4—C48	137.11 (13)	C3—C4—C48—O6	−59.04 (17)
C31—C3—C4—C48	−46.15 (15)	C5—C4—C48—O6	62.07 (16)
C3—C4—C5—C6	−13.18 (17)	C41—C4—C48—O6	−178.75 (12)
C41—C4—C5—C6	111.21 (14)	C3—C4—C48—N1	121.75 (11)
C48—C4—C5—C6	−136.05 (13)	C5—C4—C48—N1	−117.14 (11)
C3—C4—C5—C51	170.66 (11)	C41—C4—C48—N1	2.04 (13)
C41—C4—C5—C51	−64.95 (15)	C6—C5—C51—O5	178.02 (13)
C48—C4—C5—C51	47.79 (15)	C4—C5—C51—O5	−5.7 (2)
C51—C5—C6—O1	−179.49 (11)	C6—C5—C51—O4	−3.06 (18)
C4—C5—C6—O1	4.4 (2)	C4—C5—C51—O4	173.26 (11)
C51—C5—C6—C61	1.49 (19)	C5—C6—C61—C62	0.86 (19)
C4—C5—C6—C61	−174.63 (11)	O1—C6—C61—C62	−178.25 (11)
C3—C2—C21—C26	0.75 (19)	C5—C6—C61—C66	179.52 (12)
O1—C2—C21—C26	179.55 (11)	O1—C6—C61—C66	0.41 (19)
C3—C2—C21—C22	−176.13 (12)	C66—C61—C62—O4	179.57 (12)
O1—C2—C21—C22	2.68 (18)	C6—C61—C62—O4	−1.69 (19)
C26—C21—C22—C23	−1.41 (19)	C66—C61—C62—C63	−0.7 (2)
C2—C21—C22—C23	175.39 (12)	C6—C61—C62—C63	177.99 (12)
C21—C22—C23—C24	0.8 (2)	O4—C62—C63—C64	179.70 (12)
C22—C23—C24—C25	0.7 (2)	C61—C62—C63—C64	0.0 (2)
C23—C24—C25—C26	−1.5 (2)	C62—C63—C64—C65	0.7 (2)
C24—C25—C26—O2	−178.75 (12)	C63—C64—C65—C66	−0.6 (2)
C24—C25—C26—C21	0.8 (2)	C64—C65—C66—C61	−0.2 (2)
C22—C21—C26—O2	−179.85 (11)	C62—C61—C66—C65	0.8 (2)
C2—C21—C26—O2	3.10 (18)	C6—C61—C66—C65	−177.82 (12)
C22—C21—C26—C25	0.6 (2)	O6—C48—N1—C46	179.76 (13)
C2—C21—C26—C25	−176.44 (12)	C4—C48—N1—C46	−1.05 (14)
C2—C3—C31—O3	−177.26 (13)	C45—C46—N1—C48	178.55 (13)
C4—C3—C31—O3	5.88 (19)	C41—C46—N1—C48	−0.54 (15)
C2—C3—C31—O2	2.63 (18)	C5—C6—O1—C2	5.83 (18)
C4—C3—C31—O2	−174.23 (11)	C61—C6—O1—C2	−175.07 (11)
C3—C4—C41—C42	58.76 (18)	C3—C2—O1—C6	−5.26 (18)
C5—C4—C41—C42	−63.11 (17)	C21—C2—O1—C6	175.95 (11)
C48—C4—C41—C42	177.49 (13)	O3—C31—O2—C26	−178.97 (11)
C3—C4—C41—C46	−121.10 (12)	C3—C31—O2—C26	1.14 (17)
C5—C4—C41—C46	117.03 (12)	C25—C26—O2—C31	175.47 (12)
C48—C4—C41—C46	−2.37 (13)	C21—C26—O2—C31	−4.09 (18)
C46—C41—C42—C43	−0.17 (18)	O5—C51—O4—C62	−178.69 (12)
C4—C41—C42—C43	179.98 (12)	C5—C51—O4—C62	2.31 (18)
C41—C42—C43—F1	177.06 (11)	C63—C62—O4—C51	−179.61 (12)
C41—C42—C43—C44	−2.6 (2)	C61—C62—O4—C51	0.08 (19)
F1—C43—C44—C45	−177.07 (11)		

### Hydrogen-bond geometry (Å, º)

Cg1 is the centroid of the C41–C46 ring.

**Table:**

*D*—H···*A*	*D*—H	H···*A*	*D*···*A*	*D*—H···*A*
C45—H45···O5^i^	0.93	2.50	3.2564 (18)	139
C22—H22···O6^ii^	0.93	2.46	3.2205 (17)	139
C66—H66···O6^ii^	0.93	2.50	3.2921 (17)	144
C64—H64···O3^iii^	0.93	2.49	3.2641 (18)	141
C25—H25···*Cg*1^iv^	0.93	2.65	3.5482 (17)	163
N1—H1···F1^v^	0.86 (2)	2.15 (2)	2.810 (2)	134 (2)
N1—H1···O5^i^	0.86 (2)	2.51 (2)	3.208 (2)	140 (2)

Symmetry codes: (i) −*x*, −*y*+1, −*z*+1; (ii) −*x*, −*y*+1, −*z*; (iii) *x*+1, *y*+1, *z*; (iv) −*x*, −*y*, −*z*; (v) *x*−1, *y*, *z*.
